# Pharmacological treatment of pregnancy complications in adults: An overview of phase IV clinical trials

**DOI:** 10.1097/MD.0000000000041322

**Published:** 2025-01-31

**Authors:** Rawan F. Allehyani, Atheer A. Alsehli, Raghad Z. Saggat, Mohammed M. Aldurdunji, Nasser M. Alorfi

**Affiliations:** a College of Pharmacy, Umm Al-Qura University, Makkah, Saudi Arabia; b Pharmaceutical Practices Department, College of Pharmacy, Umm Al-Qura University, Makkah, Saudi Arabia; c Pharmacology and Toxicology Department, College of Pharmacy, Umm Al-Qura University, Makkah, Saudi Arabia.

**Keywords:** clinical trials, complications management, fetus health, pregnancy complications, pregnant women’s health, therapeutics

## Abstract

The aim of this review was to provide a review of the pharmacological treatments for pregnancy complications in adults. This review analyzed medications used for pregnancy complications in phase IV clinical trials based on the ClinicalTrials.gov database. The search included completed trials only. As of September 1, 2023, a total of 29,654 phase IV clinical trials were identified, of which 298 were related to pregnancy complications. Of these, 24 clinical trials met the inclusion criteria for the current study. In the 24 included clinical trials, we found 9 trials for overactive bladder with 5005 participants in total, 236 of which had adverse effects from the drugs used. Six trials for preeclampsia were conducted on 663 participants, with only 1 adverse drug effect reported. Three trials each were conducted on urinary tract infections and gestational diabetes mellitus (115 and 656 participants, respectively) without any adverse drug effects reported. One trial each focused on anemia, dystocia, and placentation disorders (80, 1003, and 14 participants, respectively) without any adverse drug effects reported. The trials reported minimal adverse drug effects, suggesting potential effectiveness and safety in managing these complications. While the trials mentioned minimal adverse effects, close monitoring and individualized patient care are essential, as are evaluating the risk–benefit ratio and the specific circumstances of each patient.

## 1. Introduction

Pregnancy is a physiological process where a female’s body undergoes significant changes to help with the development of the fetus and to support the pregnant female during pregnancy and delivery.^[1,2]^ These changes might also affect females’ perceptions of quality of life.^[3,4]^ Pregnancy causes special physiological reactions in females’ bodies that eventually lead to abnormalities in their metabolism and hormones, and their cardiovascular, musculoskeletal, and respiratory systems.^[1,5]^ These may include symptoms of nausea and vomiting, heartburn, backache, round ligament pain, frequent urination, varicose veins, constipation, leg cramps, and hemorrhoids.^[6–9]^ Those effects will be different depending on the trimester.^[10,11]^ Each trimester has different physiological changes that lead to complications and should be managed to avoid any further effects on the fetus and pregnant females.^[11,12]^ The most important aspect of the management of these complications in pregnancy is to focus on drug safety for the fetus because some medications have a teratogenic effect, which should be avoided. This review will discuss the pathophysiological changes that lead to complications during pregnancy and their pharmacological treatment.

### 1.1. Pregnancy complications

Complications arising from pregnancy consist of a range of physical and mental conditions that impact the well-being of the pregnant female and the fetus. These conditions, which can give rise to complications, may emerge prior to, during, or following pregnancy.^[10–12]^

#### 1.1.1. Genitourinary system

The genitourinary system encompasses the organs of the reproductive and urinary systems. In pregnancy, the organs of the genitourinary tract are constantly exposed to physiological changes that result in a wide variety of complications, including an OAB, infections, labor dystocia, and placentation disorders.^[13,14]^ The risk of these complications might increase in pregnancy due to the morphological and functional changes in the genitourinary tract.^[15,16]^

The effect of progesterone on pregnant women is to cause morphological and functional changes in the genitourinary tract.^[17]^ These effects include increased kidney, vagina, and uterus size; dilatation of the afferent artery of the kidney, leading to increased blood flow; and increased glomerular filtration rate (GFR), which finally leads to increased urination.^[18]^ Additionally, the uterus has an impact because it enlarges during pregnancy, increasing pressure on the bladder and increasing the frequency and urgency of urination, which may lead to OAB syndrome.^[17,19]^ Progesterone also plays a role in urinary tract infections (UTIs) because it reduces uterine motility, and an increased GFR leads to increased glucose levels in the urinary tract, increasing bacterial colonization.^[20,21]^

OAB syndrome is defined by a group of symptoms including frequent nocturnal urination, urgency, and frequency, which may impair daily life and social functioning.^[17]^ OAB pathophysiology is still unknown, but there are 2 primary theories, neurogenic and myogenic.^[14,22]^ Approximately 40% to over half of people experience bladder control problems during pregnancy.^[14]^ Also, symptoms relating to OAB during pregnancy are due to normal physiological changes during pregnancy, hormonal changes, an increase in the body mass index, and the pressure on the bladder and pelvis caused by an enlarged uterus. This occurs most frequently in the third trimester, when the fetus is at its heaviest and placing the most pressure on the bladder.^[14,18]^

The majority of OAB syndrome symptoms can be treated first with lifestyle changes such as alterations to diet, and pelvic floor muscle exercises, which are safe for pregnant females.^[23]^ The pharmacotherapy choices are anticholinergics, including oxybutynin, tolterodine, and solifenacin, which work by competitive blockage of acetylcholine at the muscarinic acetylcholine receptors.^[24,25]^ Other medication details are shown in Table 2.

Approximately 8% of pregnant females experience UTIs.^[26]^ These infections represent a spectrum, from asymptomatic bacteriuria (ASB) to symptomatic acute cystitis to the most serious, pyelonephritis.^[27]^ ASB, which affects 2% to 15% of pregnancies, is a bacterial infection of the urine without any of the typical symptoms connected with a urinary infection, such as signs of a lower urinary tract infection (acute cystitis) or an upper urinary tract/kidney infection (acute pyelonephritis).^[26,28,29]^ There are psychological reasons to predict higher rates of ASB during pregnancy, which would then increase the likelihood of the development of symptomatic UTIs and associated pregnancy problems, including increased rates of preterm delivery and low birth weight.^[26,29]^

The management of a patient with a UTI includes an initial evaluation, the selection of an antibacterial agent, the duration of therapy, and a follow-up evaluation.^[26]^ Pharmacological treatment involves antimicrobial agents with specific criteria, including being well tolerated, well absorbed, achieving high urinary concentrations, and having a spectrum of activity limited to the known or suspected pathogen.^[26,27]^ The medication is chosen according to the pathogen, to ensure that the drug covers these bacteria. Pharmacological options include trimethoprim-sulfamethoxazole, nitrofurantoin, fosfomycin, and fluoroquinolone.^[26,27,30]^ Treatments for UTI are shown in Table 1.

**Table 1:** Pharmacological treatment to manage pregnancy complications (pharmacological class, treatment and uses).

Classification	Treatment	Uses
Non-pharmacological	Lifestyle modification	Overactive bladder (OAB) syndrome
*Pharmacological class*		
Miscellaneous antibiotic	Nitrofurantoin	Urinary tract infections (UTIs)
Antifolate-sulfonamide antibiotic	Trimethoprim-sulfamethoxazole	
Phosphonic antibiotic	Fosfomycin	
Fluoroquinolones	Ciprofloxacin	
Iron replacement product	Ferrous sulphate	Iron-deficiency anemia
	Ferrous fumarate	
	Ferrous gluconate	
	Iron suspensions	
	Iron sucrose	
	Ferric carboxymaltose	
	Iron dextran	
Erythropoietin stimulating agents	Epoetin	
	Darbepoetin	
	Methoxy polyethylene glycol-epoetin beta	
Oxytocic hormones	Oxytocin	Labor dystocia
Antifibrinolytic	Tranexamic Acid	Placentation disorders
	Methotrexate *Not recommended	
Centrally acting alpha-2 adrenergic agonist	Methyldopa	Preeclampsia
Beta blocker (except atenolol)	Labetalol	
Calcium channel blocker	Long-acting nifedipine	
Vasodilator	Hydralazine	
Biguanides	Metformin	Gestational diabetes
2^nd^ generation sulfonylureas	Glyburide	
Antidiabetic hormone	Insulin	
H1 receptor antagonist (1^st^ generation)	Diphenhydramine	Hyperemesis gravidarum
Dopamine D_2_ receptor antagonist	Metoclopramide	
Serotonin 5-HT3 receptor antagonist	Ondansetron	
Phenothiazines	Promethazine	

Placentation disorders manifest in various forms and exhibit different levels of severity, depending on the specific underlying pathophysiological mechanisms involved. Placentation, a complex process influenced by prostaglandins, sexual hormones, cytokines, and immunological factors, takes place shortly after the fertilization of the ovum, and it includes placenta previa, placenta accreta, and vasa previa.^[31,32]^

Placenta accreta is characterized by excessive invasion during placenta formation, extending beyond the decidua basalis.^[31,32]^ If the placenta invades the uterine myometrium, it is referred to as placenta increta. When the placental villi penetrate the myometrium and reach the uterine serosa or adjacent organs like the bladder, it is called placenta pretreat.^[31,32]^

Placenta previa occurs when the placenta is closely attached to or covering the cervix. These conditions are associated with vaginal bleeding in the latter half of pregnancy and can cause severe complications for both the mother and the fetus, including mortality.^[31,33]^ The rates of previa and accreta are increasing, likely due to higher rates of cesarean delivery, advanced maternal age, and assisted reproductive technology.^[31,33]^

#### 1.1.2. Endocrine system

In response to glucose load in the early stages of pregnancy, pancreatic beta cells that secrete insulin experience hyperplasia, which results in elevated insulin secretion and insulin sensitivity.^[1,34,35]^ Insulin sensitivity encourages the absorption of glucose into adipose storage to meet the energy requirements until pregnancy ends.^[34]^ A glucose load also suppresses the levels of glucagon generated by the α-cells, with the greatest reduction happening at the end of the pregnancy.^[35]^

In the second trimester, the secretion of diabetogenic hormones (such as human placental lactogen, growth hormone, progesterone, cortisol, and prolactin) increases, leading to insulin resistance.^[1,34]^ That is caused by diabetogenic hormones interfering with insulin receptor signaling, which results in a reduction in insulin sensitivity in the peripheral tissues, including skeletal muscle and adipocytes.^[36,37]^ After delivery, placental hormones help with maternal insulin sensitivity, to return to prepregnancy levels within a few days.^[34]^

Gestational diabetes mellitus (GDM) arises when a female’s pancreas function is compromised and she is unable to overcome the insulin resistance that comes with pregnancy, which usually appears at the beginning of the third trimester.^[1]^ Also, GMD is a type of glucose resistance that causes hyperglycemia throughout pregnancy. Achieving glycemic control during pregnancy may offer a window of opportunity to prevent and reduce the burden of type 2 diabetes (T2D) in many generations, so any pregnant female should be screened for GDM between weeks 24 and 28 of gestation.^[1,38]^

Treatments for GDM, as shown in Table 1, consist of 3 drugs: insulin, metformin, and glyburide. Insulin is usually the first-line treatment for GDM, but if the patient has difficulties with insulin complexity or refuses the treatment, metformin and glyburide are relatively safe and effective as alternatives.^[1]^

Insulin and metformin mechanisms of action (MOA) are shown in Table 2. Glyburide MOA is that it encourages the release of insulin from beta-pancreatic cells by blocking ATP-sensitive potassium channels, leading to elevated intracellular potassium and calcium ion concentrations.^[49,50]^ It also lowers the hepatic production of glucose and increases insulin sensitivity at peripheral target sites.^[49]^

**Table 2:** Pharmacological treatments used to manage pregnancy complications.

System	Complication	Drug name	Class	MOA	Clinical uses	Side effects	Contraindication
Cardiovascular	Preeclampsia	Methyldopa	α2-adrenergic receptor agonist	Methyldopa is a centrally acting α2-adrenergic receptor agonist. It inhibits vasoconstriction via a central mechanism by reducing catecholamine release. It decreases central sympathetic outflow, decreasing systemic vascular resistance without decreasing cardiac output.^[39]^	- To treat high blood pressure, - Reduces the risk of heart attacks, kidney issues, and strokes.^[39]^	Some concern with depression, hepatic disturbances, hemolytic anemia may not lower BP adequately.^[39]^	- Active hepatic disease, - Liver disorders due to previous therapy, - Direct Coombs positive hemolytic anemia.^[39]^.
		Labetalol	Beta blockers	Labetalol reduces peripheral vascular resistance by inhibiting the adrenergic receptors, without significantly changing heart rate or cardiac output.^[40]^	- Treat angina (chest pain), - Patients with tetanus.^[40]^	- May be associated with fetal growth restriction, - Neonatal hypoglycemia with larger doses.^[40]^	- Bronchial asthma, - Greater than first degree heart block, - Severe bradycardia.^[40]^
Endocrine	Gestational diabetes	Insulin	Hormone	Insulin works by interacting directly with receptors on the cell’s plasma membrane. All cells have these receptors; however, the density of those receptors varies depending on the type of cell, with adipocytes and hepatic cells having the highest density.^[41]^	- In Type 1 or juvenile diabetes mellitus, pancreatic beta cells are destroyed by the body’s immune system or trauma or injury to the pancreas, - Type 2 diabetes mellitus, - Maturity onset diabetes of young (MODY).^[41]^	- Trembling or shaking, - Anxiety, confusion, or difficulty concentrating, - Fast heartbeat palpitations, - Tingling lips.^[42]^	- Because insulin is metabolized in the liver and eliminated in the urine, insulin dosage needs to be adjusted in individuals with renal impairment and liver failure, - In patients with a history of hypoglycemic episodes, the dosage of insulin and blood glucose levels should be closely monitored.^[41]^
		Metformin	Biguanides	- Decreases blood glucose levels by decreasing hepatic glucose production (also called gluconeogenesis), decreasing the intestinal absorption of glucose, and increasing insulin sensitivity by increasing peripheral glucose uptake and utilization.^[43]^	- Used as an adjunct to diet and exercise to improve glycemic control in adults and pediatric patients ≥ 10 years old with type 2 diabetes mellitus.^[43]^	- Abdominal or stomach discomfort, - Cough or hoarseness, - Decreased appetite, - Fast or shallow breathing, - Fever or chills, - General feeling of discomfort.^[43]^	- Congestive cardiac failure needing drug treatment, - Hypersensitivity to metformin, - Acute or chronic metabolic acidosis.^[43]^
Hematological	Anemia	Iron Sucrose (IV)	Iron replacement product	The reticuloendothelial system disassembles the compound, yielding separate entities of iron and sucrose. The liberated iron subsequently elevates serum iron concentrations and integrates into the structure of hemoglobin.^[44,45]^	- Management of cancer-associated anemia with erythropoiesis-stimulating agents.^[44]^ - Anemia in chronic kidney disease.^[44,45]^	- Gastrointestinal disorders: nausea, vomiting, constipation, diarrhea, and abdominal pain.^[44,46]^ - Skin and subcutaneous tissue disorders: pruritus, urticaria, and rash.^[47]^	- Hypersensitivity, - Non-iron-deficiency anemia, - Excess iron accumulation.^[48]^
		Ferrous Fumarate (oral)	Iron replacement product	Facilitates the substitution of iron present in hemoglobin, myoglobin, and enzymes, thereby enabling the conveyance of oxygen through the medium of hemoglobin.^[44]^	- Daily iron supplementation in adults.^[44]^ - Management of iron-deficiency anemia in adults.^[44,45]^	Like Iron sucrose.^[48]^	- Hypersensitivity, - Conditions like hemochromatosis, hemosiderosis, and hemolytic anemia (excluding cases where iron-deficiency is also present).^[45]^
Genitourinary	Labor dystocia	Oxytocin (IV)	Oxytocic hormones	The stimulation of uterine contractions is accomplished by oxytocin, which exerts its effects on receptors, such as the myometrial receptor in the fundus and the corpus. These receptors, when activated, initiate the release of calcium within the cells and facilitate the local production of prostaglandins.^[^^44,45,47^^]^	- Antepartum labor induction.^[44,45,47]^ - To control postpartum hemorrhage.^[44]^	- Water intoxication (associated with a slow oxytocin infusion over 24 h).^[44]^ - Anaphylaxis.^[44]^ - Skin and subcutaneous tissue disorders: pruritus and rash.^[46]^ - Chills and chest pain.^[46]^	The most crucial ones are: - Obstetric emergencies necessitating surgical intervention, - Fetal distress in cases where delivery is not imminent, - Contraindications to vaginal delivery, such as invasive cervical carcinoma, active herpes genitalis, total placenta previa, vasa previa, and cord presentation or prolapse, - Hyperstimulation of the uterus, characterized by strong or prolonged contractions, or a resting uterine tone between contractions of 15 to 20 mm H2O, which can result in severe complications like uterine rupture, cervical and vaginal lacerations, postpartum hemorrhage, abruptio placentae, impaired uterine blood flow, amniotic fluid embolism, and fetal trauma including intracranial hemorrhage.^[46]^
	Placentation disorders	Tranexamic Acid (IV)	Antifibrinolytics	- This compound serves as an analog of lysine and competes with lysine for binding sites on plasminogen and plasmin. Consequently, it hinders the interaction between these substances and fibrin, leading to the inhibition of fibrinolysis. Additionally, it acts to suppress the proteolytic activity of plasmin.^[44,45,47]^ Angioedema is attenuated.^[44]^	- Coronary Artery Bypass Graft Surgery.^[44,47]^ - Hereditary Angioedema.^[44]^ - Hereditary Hemorrhagic Telangiectasia.^[44]^ - Life-Threatening Hemorrhage.^[44]^ - Perioperative Prevention of Blood Loss and Transfusion, Orthopedic Surgery.^[44,45]^ - Postpartum Hemorrhage.^[44]^ - Surgical and procedural management.^[44]^	- Gastrointestinal disorders: Nausea, vomiting, and abdominal pain.^[^^44^^]^ - Nervous system disorders: dizziness, and headache.^[^^44^^]^ - Chills and chest pain. - Skin and subcutaneous tissue disorders: pruritus and rash.	- Hypersensitivity, - Acquired defective color vision, - Subarachnoid hemorrhage, - Active intravascular clotting.^[48]^
	Urinary incontinence	OnabotulinumtoxinA	Neuromuscular blocker agent	Prevents calcium-dependent release of acetylcholine and produces a state of denervation.^[44]^	- Neurogenic lower urinary tract dysfunction, - Diagnosis and treatment of overactive bladder (non-neurogenic) in adults.^[44]^	- Bladder dysfunction, - Cervical dystonia, - Upper limb spasticity.^[44]^	Hypersensitivity to any botulinum toxin preparation or any component of the formulation.^[44]^
		Microbiota	Microbiota Beta-3 agonist	Restoration of intestinal eubiosis.^[44]^	*C. difficile* infection.^[44]^	–	Severe hypersensitivity (e.g., anaphylaxis) to fecal microbiota (live) or any component of the formulation.^[44]^
	Overactive bladder (OAB)	Mirabegron	Beta-3 agonist	Activates beta-3 adrenergic receptors in the bladder resulting in relaxation of the detrusor smooth muscle during the urine storage phase, thus increasing bladder capacity.^[45]^	- Benign prostatic hyperplasia, - Overactive bladder.^[45]^	- Cardiovascular: hypertension and tachycardia - Gastrointestinal: abdominal pain, constipation, and diarrhea (2%), - Genitourinary: cystitis ^[45]^.	Hypersensitivity to mirabegron or any component of the formulation.^[45]^
		Tolterodine	Anticholinergic agents	Competitive antagonist of muscarinic receptors.^[44]^	- Benign prostatic hyperplasia - Overactive bladder ^[45]^.	Gastrointestinal: xerostomia Cardiovascular: Chest pain Central nervous system: headache, dizziness, and anxiety.^[45]^	Hypersensitivity to tolterodine or fesoterodine.^[44]^
		Trospium		Antagonizes the effects of acetylcholine on muscarinic receptors in cholinergically innervated organs.	- Benign prostatic hyperplasia, - Overactive bladder.	- Gastrointestinal: xerostomia - Cardiovascular: Tachycardia - Central nervous system: headache.	Hypersensitivity to trospium or any component of the formulation; patients with or at risk of urinary retention, gastric retention, uncontrolled narrow-angle glaucoma.
		Solifenacin		Inhibits muscarinic receptors resulting in decreased urinary bladder contraction, increased residual urine volume, and decreased detrusor muscle pressure.^[44]^	- Benign prostatic hyperplasia - Overactive bladder.^[44]^	- Gastrointestinal: constipation - Cardiovascular: hypertension.^[44]^	Hypersensitivity (e.g., anaphylaxis, angioedema) to solifenacin or any component of the formulation; urinary retention gastric retention; uncontrolled narrow-angle glaucoma.^[45]^
		Fesoterodine		Antagonist of muscarinic receptors.^[44]^	- Benign prostatic hyperplasia - Overactive bladder.^[44]^	- Gastrointestinal: diarrhea - Cardiovascular: peripheral edema - Dermatological: skin rash.^[44]^	Hypersensitivity (e.g., angioedema) to fesoterodine, tolterodine, or any component of the formulation; urinary retention; gastric retention; uncontrolled narrow-angle glaucoma.^[44]^

#### 1.1.3. Cardiovascular system

Pregnancy causes major physiological changes to the cardiovascular system, including increased heart rate, increased stroke volume, increased cardiac output, and decreased vascular resistance.^[1]^

In pregnancy, uterine spiral arteries normally develop into high-capacity blood vessels (vasodilation). This process is defective in patients with preeclampsia.^[51]^ The abnormal spiral arteries have vasocontraction, which affects the transformation of nutrition to the fetus. It also affects the mother by secreting a pro-inflammatory that affects the blood vessels, leading to systemic vasoconstriction and organ dysfunction, which as a consequence leads to an increase in blood pressure, known as preeclampsia.^[51,52]^

Preeclampsia is a serious medical condition that can develop during pregnancy, usually after the 20th week, and less commonly in the postpartum period. It is characterized by hypertension (HTN), systolic blood pressure (SBP) ≥ 140 mm Hg, and/or diastolic blood pressure (DBP) ≥ 90 mm Hg, along with the presence of protein in the urine (proteinuria), which indicates kidney involvement and damage. This leads to multiple organ damage, most commonly damage in the liver and kidneys.^[51,53]^

Treatments for preeclampsia, as shown in Table 2, are labetalol, long-acting nifedipine, methyldopa, and hydralazine.^[1]^ Labetalol, nifedipine, and methyldopa are the first lines of treatment during pregnancy; calcium channel blockers (CCB) and other beta blocker drugs (except atenolol) are considered alternatives.^[1]^

As a CCB, nifedipine lowers arterial blood pressure by reducing peripheral vascular resistance, which is caused by blocking calcium ions from entering certain voltage-sensitive areas of the myocardium and vascular smooth muscle during depolarization.^[39,40]^ This relaxation of the coronary vascular smooth muscle and coronary vasodilation results from this inhibition.^[39,40]^ Labetalol and methyldopa MOA are shown in Table 2.

#### 1.1.4. Hematological

Hemoglobin’s primary role is to carry oxygen to tissues and eliminate carbon dioxide from the lungs.^[41]^ A fall in hemoglobin levels is observed during pregnancy in healthy females who are not deficient in iron or folate. This is caused by a relatively greater expansion of plasma volume compared with the increases in hemoglobin mass and red blood cell volume that accompany a normal pregnancy.^[41,42]^ Early in pregnancy, the hemoglobin level of most healthy females with iron stores is 11 g/dL or higher.^[43]^

The objectives of treatment are the correction of the deficit in hemoglobin mass and eventually the restitution of iron stores.^[48]^ Both objectives can be accomplished with orally administered simple iron compounds such as ferrous sulfate, fumarate, or gluconate, which provide a daily dose of approximately 200 mg of elemental iron.^[48]^ To replenish iron stores, oral therapy should be continued for 3 months or so after the anemia has been corrected. Transfusions of red blood cells or whole blood are indicated for the treatment of iron-deficiency anemia unless hypovolemia from blood loss co-exists, or an emergency operative procedure must be performed on a severely anemic female (hematocrit <20 volume percent).^[44]^ MOAs are shown in Table 2.

#### 1.1.5. Gastrointestinal system

There are several theories related to the cause of gastrointestinal complications. They appear to be related to hormonal changes in the levels of human chorionic gonadotropin (hCG) and estrogen.^[46]^ Those changes may lead to hyperemesis gravidarum or gastroesophageal reflux disease (GERD)^[47]^

Hyperemesis gravidarum is a severe type of vomiting in pregnancy that has a huge effect on a pregnant female’s health. It may cause hypokalemia, acidosis from malnutrition, alkalosis from the loss of hydrochloric acid in the vomitus, dehydration, and weight loss.^[47]^

It is commonly recognized that increases in progesterone and estrogen levels cause the lower esophageal sphincter to relax, leading to GERD, which begin with nausea.^[46]^ Treatment usually includes non-pharmacological options like eating small amounts at more frequent intervals and avoiding starvation.^[45]^ It is also recommended to avoid foods that precipitate or aggravate symptoms. Pharmacological treatments are shown in Table 1.

Pregnancy-related changes in gastrointestinal motility make gastroesophageal reflux illness almost universal.^[45,54]^ Non-pharmacological treatment options include lifestyle modification (small frequent meals, avoiding meals prior to bedtime, elevating the head of the bed, and avoiding smoking, caffeine, and alcohol).^[45,54]^ Pharmacological treatments are shown in Table 1.

## 2. Methods

### 2.1. Search source

We conducted a search on ClinicalTrials.gov to locate trials applicable to this review. ClinicalTrials.gov serves as an internet-based database, functioning as a publicly accessible registry for clinical trials carried out in 221 countries. It provides comprehensive information regarding medical studies involving human volunteers.

### 2.2. Search criteria

**Figure 1.**
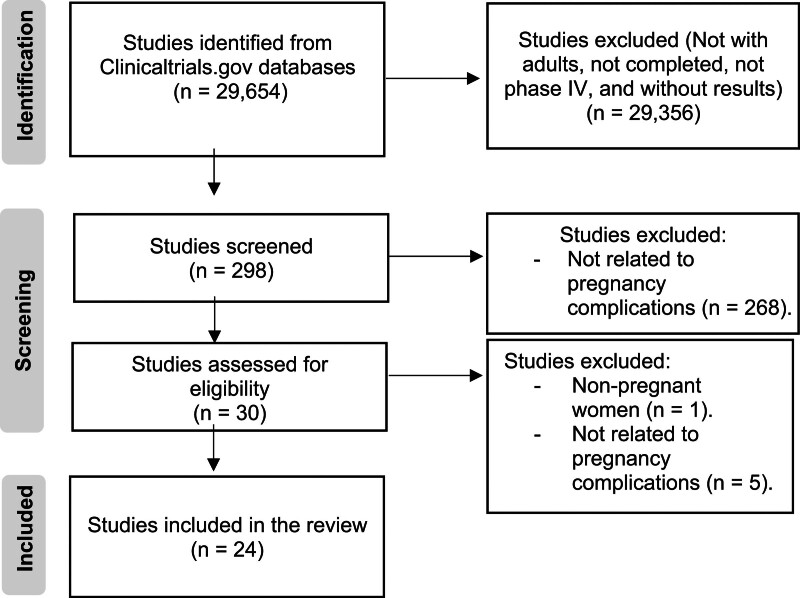
Flow chart of clinical trials selection process.

In this review, an assessment was conducted on medications utilized for specific pregnancy complications. The focus was on examining entries associated with pharmacological studies related to pregnancy complications. Flow chart of clinical trials selection process shown at Figure 1.

### 2.3. Study design

The search parameters used in the study can be found in the subsequent section. Four reviewers independently screened the articles and evaluated them for potential bias. On September 1, 2023, a total of 29,654 clinical trials data were obtained from ClinicalTrials.gov. Following the exclusion of trials focused on treating different complications or those that were not in phase IV, a subset of 24 clinical trials remained eligible for inclusion in the review. The criteria for inclusion in this study were as follows:

1. Primary focus on pregnancy complications.
2. Completed study.
3. Involving adult patients within the age range of 18 to 64 years old.
4. Available results.
5. Limited to female participants.

### 2.4. Data extraction

The data extraction process involved manual retrieval and download of the relevant information from ClinicalTrials.gov, encompassing the following aspects:

Conditions: The medical condition treated was selected as “pregnancy complications.”
Trial design: Phase IV only.

The results of the trials were manually extracted from the reported outcomes within the registry. The primary outcomes, number of participants, timeframe, and results were collected as part of the data extraction process.

## 3. Results

**Figure 2.**
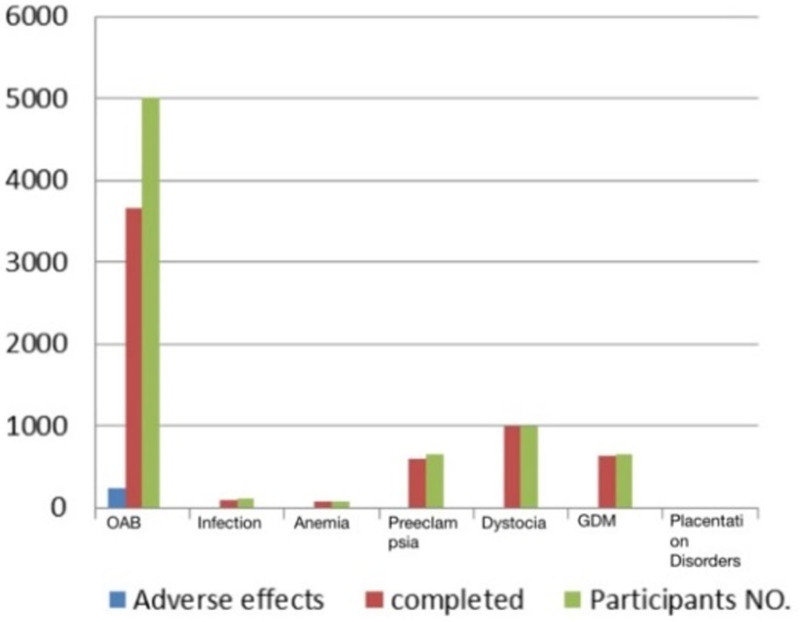
ClinicalTrials.gov result bar graph.

**Figure 3.**
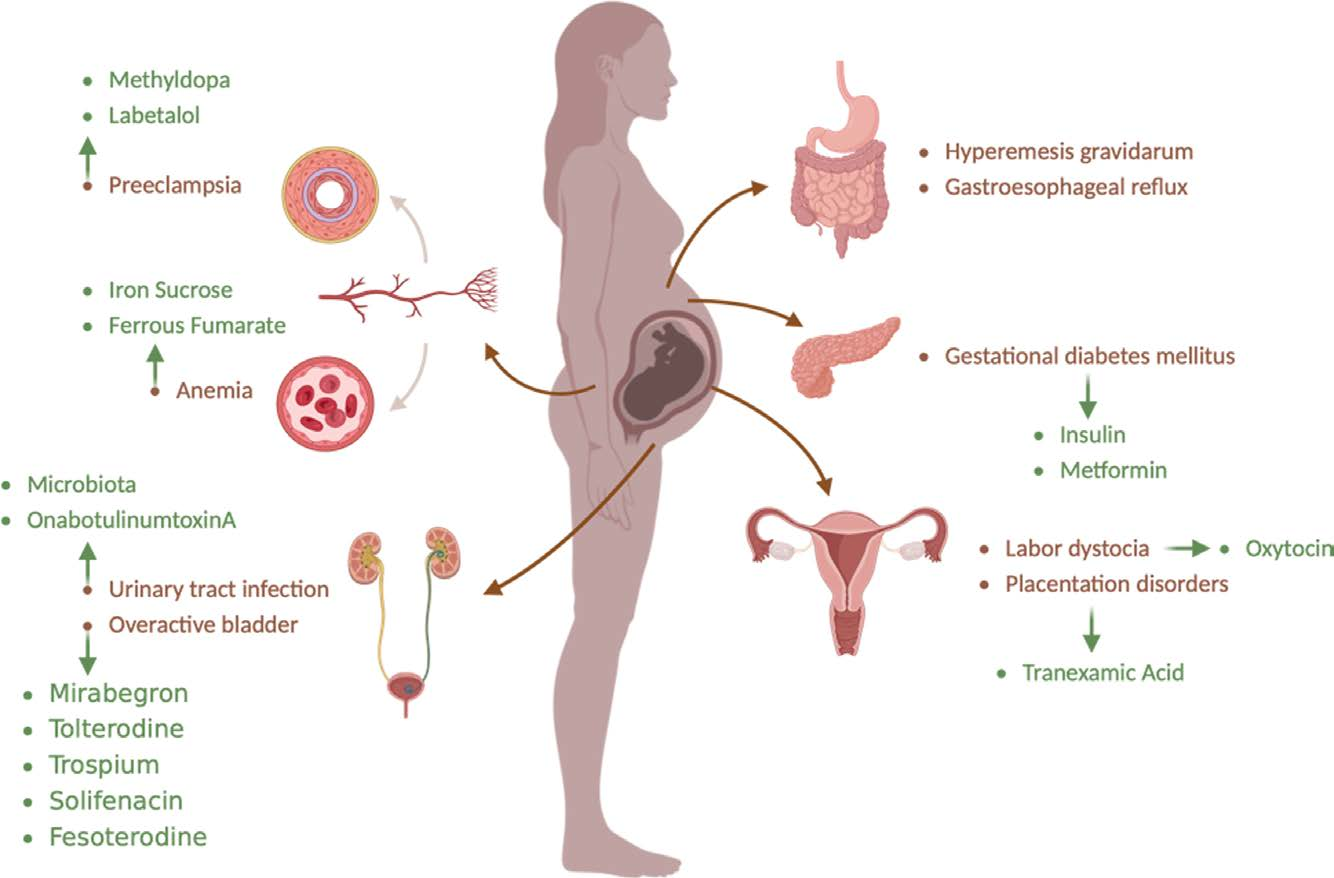
Pregnancy complications and their ClincalTrials.gov managements.

In the 24 included clinical trials, we found 9 trials for OAB with 5005 participants in total, 236 of which experienced adverse effects from the drugs used. Also, for preeclampsia, 6 trials were conducted on 663 participants, with only 1 adverse drug effect reported. Three trials each were conducted on urinary tract infection and GDM (115 and 656 participants, respectively) without any adverse drug effects reported. Lastly, 1 trial was performed on each of IDA, dystocia, and placentation disorders (80, 1003, and 14 participants, respectively) without any adverse drug effects reported. All studies are shown in Table 2 and Figure 2. A summary of the complications and their medications according to ClinicalTrials.gov is shown in Figure 3.

### 3.1. Overactive bladder results

Nine randomized trials were found for treating an OAB during pregnancy. The first trial measured the change from baseline in the daily average number of urinary incontinence episodes as a primary outcome, comparing treatment with onabotulinumtoxinA and a placebo. In total, 78 participants were in the onabotulinumtoxinA group and 39 were in the placebo group. There was a statistically significant *P*-value = <.0001.

The second trial measured the participants’ tolerance of mirabegron and tolterodine ER. This was assessed by the medication tolerability scale of the overactive bladder-satisfaction (OAB-S) questionnaire at the end of treatment (EOT) as a primary outcome. A total of 341 participants were allocated to the mirabegron group and 336 to the tolterodine ER group. There was a statistically significant *P*-value = 0.004.

The third trial measured the number of participants with treatment-emergent adverse events (TEAEs) as a primary outcome when comparing treatment with mirabegron + solifenacin; treatment with mirabegron + propiverine; treatment with mirabegron + imidafenacin; and treatment with mirabegron + tolterodine. The total numbers of participants analyzed were 166, 161, 161, and 159, respectively, with TEAEs numbers of 131, 135, 133, and 120, respectively.

The fourth trial measured the percentage of participants who achieved a 100% reduction in urinary incontinence episodes. A change from the baseline in the daily average number of episodes of urinary incontinence was the primary outcome, comparing treatment with onabotulinumtoxinA and a placebo. There were 128 participants in the onabotulinumtoxinA group and 125 in the placebo group. The study reported the mean of change at week 12 as −3.4 in the onabotulinumtoxinA group and −1.7 in the placebo group.

The fifth trial employed Hopkins Verbal Learning Test to assess short-term verbal learning and memory as a primary outcome when taking trospium chloride and a placebo. There were 21 participants in the trospium chloride group and 24 in the placebo group. The mean scores were 81.68 in the trospium chloride group and 83.49 in the placebo group.

The sixth trial measured the number of participants with TEAEs as the primary outcome, comparing treatment with solifenacin 2.5 mg + mirabegron 25 mg; solifenacin 2.5 mg + mirabegron 50 mg; solifenacin 5 mg + mirabegron 25 mg; and solifenacin 5 mg + mirabegron 25 mg. The total numbers of participants analyzed in each group were 35, 37, 58, and 93, respectively, with numbers of TEAEs of 23, 21, 42, and 69, respectively.

The seventh trial measured the change from baseline in the mean number of urgency urinary incontinence (UUI) episodes per 24 hours at week 12 as a primary outcome, comparing treatment with fesoterodine 4 mg and a placebo. The total numbers of participants analyzed were 733 in the fesoterodine 4 mg group and 370 in the placebo group. The least squares mean was −2.85 in the fesoterodine 4 mg group and −2.2 in the placebo group.

The eighth trial measured the mean number of urgency urinary incontinence (UUI) episodes per 24 hours as a primary outcome, comparing treatment with fesoterodine: double-blind baseline and a placebo. The overall number of participants analyzed was 292 in the fesoterodine: double-blind group and 279 in the placebo group. There was a statistically significant *P*-value = 0.0079.

The ninth trial measured the percentage of patients’ continent (PPC) as a primary outcome, comparing treatment with sanctura XR® and a placebo. The total numbers of participants analyzed were 156 in the sanctura XR® group and 152 in the placebo group. The PPC was 34.6 in the sanctura XR® group and 17.1 in the placebo group.

### 3.2. Dystocia results

With regard to treating dystocia, 1 trial was conducted to test oxytocin’s efficacy. The trial enrolled 1003 participants and divided them into 2 similar-sized groups (a high-dose oxytocin regimen group and a low-dose oxytocin regimen group). The primary outcome was the number of babies delivered by cesarean, which was 73 (14.5%) in the high-dose oxytocin group and 72 (14.4%) in the low-dose oxytocin group.

### 3.3. Preeclampsia results

Six randomized trials were performed on treating preeclampsia. The first trial enrolled 110 participants, with 55 allocated to the intervention group (oral nifedipine 30 mg) and 55 to the control group (placebo). The primary outcome was the number of participants with a change in blood pressure (both systolic and diastolic). In the intervention group, 53 participants were analyzed, and 18 (34%) had a change in blood pressure. In the control group, 49 participants were analyzed, and 27 (55.1%) had a change in their blood pressure.

The second trial on preeclampsia compared treatment with oral nifedipine and IV labetalol. The study enrolled 109 participants; 54 were in the nifedipine group and 55 in the labetalol group. The first primary outcome was the time to achieve a non-severe range of blood pressure measured by the median (IQR). In the nifedipine group, this was 10 minutes (10–20), and in the labetalol group, this was 20 minutes (20–30). As for the second primary outcome, which was the number of participants to achieve non-severe range blood pressure numbers, it was as follows: 32 White, Asian, and Hispanic participants were analyzed in the nifedipine group and 31 (96.9%) achieved the outcome. In the labetalol group, 35 were analyzed and 33 (94.3%) achieved the outcome. Twenty-two Black participants were analyzed in the nifedipine group and all achieved the outcome; and 20 participants were analyzed in the labetalol group and 18 (90%) achieved the outcome.

The third trial on preeclampsia assessed the efficacy of Isosorbide mononitrate. It enrolled 176 participants, 89 in the intervention group and 87 in the control group. The primary outcome was the cesarean delivery rate, which was 29 participants (32.6%) in the intervention group and 22 participants (25.3%) in the control group.

The fourth trial on preeclampsia compare the efficacy of nifedipine and enalapril. In total, 94 participants were enrolled and divided into 2 groups (the nifedipine group and the enalapril group). The first primary outcome was the number of participants who had prolonged hospitalization, which was 13 participants (27.7%) in the nifedipine group and 17 participants (36.2%) in the enalapril group. As for the second primary outcome, the number of participants who had an unscheduled clinic appointment was 19 (40.4%) in the nifedipine group and 17 (36.2%) in the enalapril group.

The last 2 trials on preeclampsia compared acetaminophen and ibuprofen. The first trial enrolled 100 participants, divided equally into 2 groups (the acetaminophen group and the ibuprofen group). The primary outcome was the duration of severe-range hypertension after delivery. All the participants were analyzed and the result was 38 hours (CI 95%, were 29.4 to 51.3) in the acetaminophen group and 35.3 hours (CI 95% was 27.2–47.5) in the ibuprofen group; *P* = .3. The second trial enrolled 74 participants divided between the same 2 treatment groups. The primary outcome was the difference in systolic blood pressure (SBP) 24 hours following the intervention, measured as the mean (SD), which was −2.7 (8.1) in the ibuprofen group and −2.9 (8.6) in the acetaminophen group.

### 3.4. Anemia results

A randomized open-label trial was conducted on treatment for anemia in pregnancy. The difference in elevating serum ferritin levels was measured as a primary outcome between the IV and oral iron groups. This trial enrolled 80 participants: 40 on IV iron sucrose and 40 on oral ferrous fumarate. The serum ferritin level was measured 3 weeks after the intervention. The overall number of participants analyzed was 38 on IV iron sucrose with a mean (SD) change of 136.1 µg/dL (55) in serum ferritin levels, and 36 on oral ferrous fumarate with a mean (SD) change of 28.3 µg/dL (14.8) in serum ferritin levels.

### 3.5. Gestational diabetes results

The study of gestational diabetes evaluated the safety and efficacy of Humalog Mix 50/50TM administered as 3 injections daily, compared to Humalog Plus Humulin N insulin administered as 6 separate injections daily for women with GDM. The primary outcome measure was the comparison of hemoglobin A1C levels between the 2 treatment groups. Twenty participants were analyzed in each treatment group. The mean hemoglobin A1C level was 5.5% with a standard deviation of 0.3% in the Humalog Mix50/50TM group and 5.6% with a standard deviation of 0.3% in the Humalog Plus Humulin N group.

The second trial on gestational diabetes investigated whether the use of an oral hypoglycemic agent, glyburide, as an adjunct to diet therapy in women with mild GDM, achieved euglycemia in a shorter period of time and reduced the frequency of maternal and neonatal morbidities. The primary outcome measure was the mean weight at birth, assessed immediately after the delivery of the baby. A total of 186 participants were analyzed in the placebo group, and 189 participants were analyzed in the glyburide group. The mean weight at birth was 3355 grams with a standard deviation of 521 grams in the placebo group and 3322 grams with a standard deviation of 481 grams in the glyburide group. The study results indicate that there was no significant difference in mean weight at birth between the glyburide and placebo groups.

The third trial on gestational diabetes compared the efficacy of metformin versus insulin for the treatment of GDM. It included a total of 217 women, with 110 receiving metformin and 107 receiving insulin. The primary outcome measure was birth weight adjusted for gestational weeks, expressed as standard deviation units using data from Finnish fetal growth charts for normal pregnancies. Birth weight was assessed at the time of delivery. In the metformin group, the mean birth weight was 3604 grams, with a standard deviation of 488 grams. In the insulin group, the mean birth weight was 3589 grams with a standard deviation of 448 grams. The study findings suggest that there was no significant difference in birth weight between infants born to mothers treated with metformin compared to those treated with insulin for gestational diabetes.

### 3.6. Urinary tract infection results

The study of subclinical bacteriuria in pregnancy investigated whether treating pregnant women who had urine cultures showing low levels of bacteria (<100,000 CFU) could reduce adverse pregnancy outcomes, particularly the incidence of cystitis. Cystitis was defined as a urine culture with more than 100,000 CFU at any point during antenatal care. The primary outcome measure was the number of participants who developed cystitis during the study period, which lasted about 10 months. In the “No Antibiotic Treatment” arm, out of 28 participants, 14.3% developed cystitis. In the “Antibiotic Treatment” arm, 4 out of 25 participants (16.0%) developed cystitis.

The second trial on urinary tract infection provided insights into the effects of estrogen treatment on pelvic floor microbiome (PFM) diversity, female urinary microbiota (FUM), antimicrobial peptide (AMPs) levels, and OAB symptoms in hypoestrogenic women. The primary outcome of the study was to measure the change in the relative abundance of Lactobacillus in the vaginal microbiome before and after treatment with estrogen cream (Premarin Cream®) over a period of 12 weeks. Participants who received estrogen cream (Premarin Cream®) showed a mean change in the relative abundance of Lactobacillus of 0.275 (standard deviation: 0.345). This indicates an increase in the proportion of Lactobacillus in the vaginal microbiome after 12 weeks of treatment with estrogen cream.

The third trial on infection measured the change in inflammatory markers in urine using immunoenzyme assays after standard-of-care anticholinergic treatment for urgent urinary incontinence. The primary outcome of the study involved testing the difference in inflammatory markers between baseline and follow-up using the Wilcoxon rank sum test. P-values for all inflammatory markers were provided, indicating the probability that the observed differences between baseline and follow-up levels were due to chance. None of the p-values reached statistical significance, suggesting no significant difference in inflammatory markers between baseline and follow-up after standard-of-care anticholinergic treatment.

### 3.7. Placenta disorder results

The study on placenta previa and placenta accreta investigated the effectiveness of intravenous tranexamic acid (TXA) in reducing blood loss during high-risk surgical procedures related to placenta previa and placenta accreta. The primary outcome measure was the estimated blood loss (EBL), which was assessed 1 to 3 hours post-surgery. In the treatment arm (TXA group), the mean EBL was 3116 cc with a standard deviation of 3947 cc. In contrast, in the placebo arm, the mean EBL was 9420 cc with a standard deviation of 12474 cc. The results suggest a potential benefit of intravenous TXA in reducing blood loss during high-risk obstetric surgeries related to placenta previa and accreta, compared to the placebo group.

## 4. Discussion

The review analysis assesses the phase IV clinical trials of pregnancy complications based on the ClinicalTrials.gov database, which showed a well-correlated relationship between the pathology of disease incidence and normal physiological changes that occurred during pregnancy. These pregnancy-related complications may cause alterations in various aspects of pregnant females’ health.

Based on the inclusion criteria, pregnancy complications were found in 24 cases. These included dystocia, preeclampsia, GDM, anemia, and urinary incontinence. The ideal time for the correction of underlying complications is before delivery, and these women should be properly followed. As per the guidelines, most of the women were successfully treated by conservative management, such as treating the underlying medical problems. Also, an essential aspect to consider when using these medications for managing pregnancy complications is the potential occurrence of side effects. While the trials reported minimal adverse drug effects, it is crucial to closely monitor patients during treatment. Each medication carries its own potential side effects, and healthcare professionals should be involved in assessing and responding to any adverse reactions that may arise.

Furthermore, it is essential to highlight the importance of individualized patient care and the consideration of risk–benefit ratios when prescribing these medications. Pregnancy is a sensitive period, and the potential impact of any medication on both the mother and the baby must be completely evaluated. Healthcare providers should carefully assess the potential risks and benefits associated with these pharmacological interventions, considering the specific circumstances and health status of each patient.

## 5. Conclusion

Physiological changes that occur during pregnancy are the primary cause of most pregnancy complications. A summary of some of the pharmacological treatments for complications in the genitourinary, endocrine, cardiovascular, hematological, and gastrointestinal tract systems was provided in this article. Mainly, maintaining a healthy lifestyle during pregnancy would help with or prevent most of the complications. The trials reported minimal adverse drug effects, suggesting potential effectiveness and safety in managing these complications, but close monitoring and individualized patient care are still essential, as are evaluating the risk–benefit ratio and considering the specific circumstances of each patient.

## Author contributions

**Conceptualization:** Rawan F. Allehyani, Atheer A. Alsehli, Raghad Z. Saggat.

**Data curation:** Rawan F. Allehyani, Atheer A. Alsehli, Raghad Z. Saggat.

**Funding acquisition:** Nasser M. Alorfi.

**Investigation:** Atheer A. Alsehli, Raghad Z. Saggat.

**Methodology:** Raghad Z. Saggat, Mohammed M. Aldurdunji.

**Resources:** Mohammed M. Aldurdunji.

**Supervision:** Nasser M. Alorfi.

**Validation:** Atheer A. Alsehli, Raghad Z. Saggat.

**Visualization:** Atheer A. Alsehli, Raghad Z. Saggat.

**Writing – original draft:** Nasser M. Alorfi, Rawan F. Allehyani, Atheer A. Alsehli, Raghad Z. Saggat.

**Writing – review & editing:** Nasser M. Alorfi, Rawan F. Allehyani, Atheer A. Alsehli, Raghad Z. Saggat.
